# Warming Can Boost Denitrification Disproportionately Due to Altered Oxygen Dynamics

**DOI:** 10.1371/journal.pone.0018508

**Published:** 2011-03-31

**Authors:** Annelies J. Veraart, Jeroen J. M. de Klein, Marten Scheffer

**Affiliations:** Department of Aquatic Ecology and Water Quality Management, Wageningen University, Wageningen, The Netherlands; Netherlands Institute of Ecology, Netherlands

## Abstract

**Background:**

Global warming and the alteration of the global nitrogen cycle are major anthropogenic threats to the environment. Denitrification, the biological conversion of nitrate to gaseous nitrogen, removes a substantial fraction of the nitrogen from aquatic ecosystems, and can therefore help to reduce eutrophication effects. However, potential responses of denitrification to warming are poorly understood. Although several studies have reported increased denitrification rates with rising temperature, the impact of temperature on denitrification seems to vary widely between systems.

**Methodology/Principal Findings:**

We explored the effects of warming on denitrification rates using microcosm experiments, field measurements and a simple model approach. Our results suggest that a three degree temperature rise will double denitrification rates. By performing experiments at fixed oxygen concentrations as well as with oxygen concentrations varying freely with temperature, we demonstrate that this strong temperature dependence of denitrification can be explained by a systematic decrease of oxygen concentrations with rising temperature. Warming decreases oxygen concentrations due to reduced solubility, and more importantly, because respiration rates rise more steeply with temperature than photosynthesis.

**Conclusions/Significance:**

Our results show that denitrification rates in aquatic ecosystems are strongly temperature dependent, and that this is amplified by the temperature dependencies of photosynthesis and respiration. Our results illustrate the broader phenomenon that coupling of temperature dependent reactions may in some situations strongly alter overall effects of temperature on ecological processes.

## Introduction

**Figure 1:**
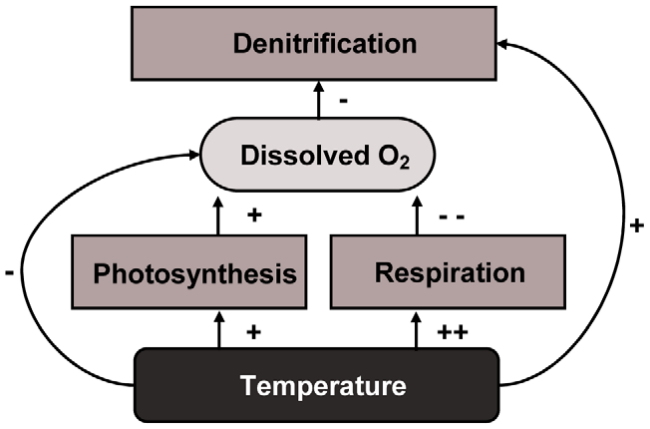
Schematic overview of major direct and indirect effects of temperature on denitrification.

Anthropogenic activities have greatly increased reactive nitrogen inputs to aquatic ecosystems, which has led to numerous eutrophication problems such as harmful phytoplankton blooms, temporal hypoxia and fish death [1]. Denitrification is the main nitrogen removing process in freshwater ecosystems, it reduces nitrate to gaseous nitrogen under anoxic conditions [2]. Effects of climate change on denitrification have been difficult to predict because of the complex of biogeochemical interactions involved [3]. Predicting these effects for aquatic ecosystems is even more difficult as data on the effects of temperature on denitrification in aquatic ecosystems are sparse [4]. As most biochemical reactions occur at higher rates when temperature increases [5] we expect increased denitrification rates at elevated temperatures. However, the intensity of the impact of temperature on denitrification rates appears to vary widely between systems [2], [3]. In anaerobic soil slurries and batch reactors under controlled conditions [6], [7] denitrification shows only a moderate effect of temperature, whereas a study in constructed wetlands shows much stronger temperature effects [8]. This suggests that the strong temperature dependence of denitrification might arise from coupled temperature dependent processes that are excluded in the bioreactors but captured in more natural environmental settings. As denitrification is strongly affected by oxygen levels, we explored whether the direct effect of temperature on denitrification could be amplified by a temperature dependence of dissolved oxygen concentrations. Temperature affects dissolved oxygen concentration (DO) in aquatic ecosystems in different ways. Solubility of oxygen in water decreases with temperature, and high temperatures also tend to promote respiration more than photosynthesis [9], potentially implying a decrease of DO beyond the solubility effect. The resulting drop in oxygen could boost denitrification rates (Fig. 1). We tested this idea by analyzing field data on denitrification, temperature and DO, in combination with lab experiments assessing the effect of temperature on DO and the effect of DO on denitrification separately. In addition, we used a simple model to further explore if the temperature effect on denitrification can be realistically explained by the coupled temperature dependencies of respiration, primary production and oxygen solubility.

## Results and Discussion

**Figure 2:**
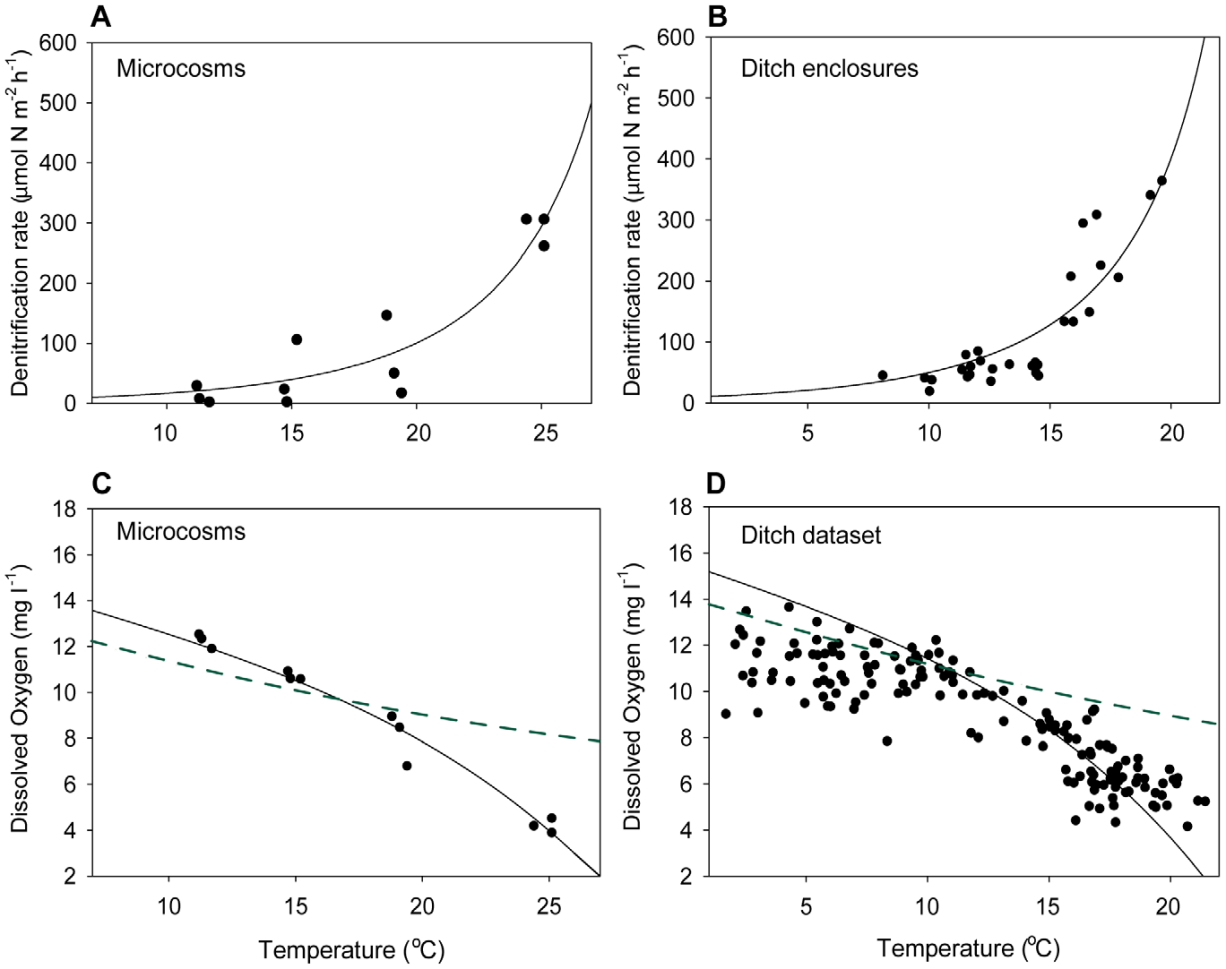
Temperature dependence of denitrification and dissolved oxygen concentrations. Panels show: Denitrification rates at different temperatures in vegetated microcosms (A) and vegetated drainage ditches (B). Dissolved oxygen concentrations at different temperatures in vegetated microcosms (C) and in drainage ditches based on monthly average values (April-July) at 3100 sites for the years 1980–2005 (D). Solid lines show the model predictions based on equation (2) (panels A and B), and equation (3) (panels C and D). Dashed lines represent the temperature dependence of the saturation concentration of oxygen (equation 4).

We found an exponential increase of denitrification with temperature in both the microcosms and in the field (Fig. 2 A, B). The overall temperature effect on denitrification could be quantified by a modified Arrhenius expression [10]:
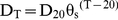
(1)where D_T_ is the denitrification rate in µmol N m^−2^ h^−1^, at temperature T (°C), D_20_ is the denitrification in µmol N m^−2^ h^−1^ at 20°C, and θ_s_ is the overall system temperature coefficient (dimensionless) [5], [10]. For most biochemical reactions in this temperature range reaction rates double with a ten degree temperature increase, which corresponds to a θ_s_ of around 1.07 (Q_10_ = θ^10^) [10]. We observed a stronger temperature response of denitrification, with temperature coefficients θ_s_ with a value of 1.24 in the microcosms (*n* = 12), and 1.28 in the ditch enclosures (*n* = 29). This means that a one degree temperature rise led to 24 to 28 percent higher denitrification rates. These temperature effects resemble those found in constructed wetlands [8]. However, they are nearly three times stronger, in terms of percent increase, than those found in controlled batch reactors and anoxic soil slurries, where temperature coefficients ranged from 1.06 to 1.13 [6], [7], [11], [12].

Besides the effect of temperature on denitrification, this experiment also clearly showed a temperature dependence of DO (Fig. 2C). As predicted, oxygen levels dropped faster with rising temperatures than can be expected from reduced solubility alone. A similar temperature dependence of DO was observed in a dataset containing 100 monthly averages of DO and temperature (April-July) for all years in the period 1980–2005, based on measurements in 3100 ditches throughout the Netherlands (Fig. 2D). These results indicate that indeed respiration is more strongly affected by temperature than photosynthesis, as has also been found in other studies [9]. The fundamentally different temperature dependencies of photosynthesis (in primary producers) and respiration (on all trophic levels) are determined by their specific enzymatic temperature dependencies. Photosynthesis is constrained by the temperature dependence of Rubisco carboxylation in the chloroplasts [9], whereas respiration is constrained by the temperature dependence of ATP synthesis in respiratory complexes [9], [13].

**Figure 3:**
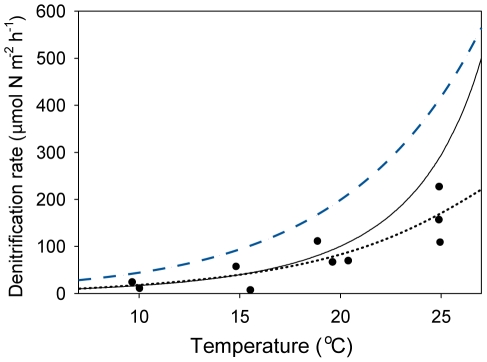
Temperature dependence of denitrification rates at constant low dissolved oxygen levels. Denitrification rates measured in vegetated freshwater microcosms, as compared to the fitted Arrhenius equation (dotted black line; equation 1), the modelled denitrification rates (black line; equations 2–4 with parameters from Table 2), and modelled denitrification with O_2_ fixed at 1 mg l^−1^(dashed blue line; equation 2).

To test if this temperature dependence of DO can explain the strong temperature dependence of denitrification we performed a second microcosm experiment in which we excluded the oxygen effect. Keeping DO constant and low (0.6–1 mg l^−1^) we found a temperature coefficient θ_s_ for denitrification of only 1.15 (*n* = 12, Fig. 3, Table 1), which was substantially lower than the temperature coefficient observed in the previous experiment in which DO was left free to vary with temperature. These results indicate that the temperature dependence of DO may indeed boost the effect of temperature on denitrification. Our simple model approach supported this finding, using parameters from the literature and our experiments (see methods), the model could reproduce the experimental data well (Fig. 2 A, B) (equation 2, R^2^ = 0.86). Non-linear regression using equation (1) on the modelled data yielded an estimated temperature coefficient θ_s_ of 1.30 (R^2^ = 0.995). By contrast, the model predicted a much lower temperature coefficient for denitrification if we mimicked a situation in which oxygen was unaffected by temperature by keeping DO at 1 mg l^−1^. The resulting temperature dependence of denitrification (θ_s_ = 1.16, Table 1) is in good agreement with the temperature coefficient of 1.15 for denitrification at fixed oxygen concentrations that we found in the corresponding experiment. Thus the model confirms that the observed effects of temperature on denitrification may reasonably be explained by correlated temperature effects on DO.

**Table 1:** Overall temperature effects on denitrification (equation 1) with either temperature dependent or fixed dissolved oxygen levels.

Set-up	DO	D_20_	Overall θ_s_	R^2^	≈Q_10_
Microcosm	T dependent	100.6	1.24	0.87	9
Field	T dependent	430	1.28	0.79	12
Microcosm	Fixed (0.6–1 mg l^−1^)	81.2	1.15	0.74	4
Model	T dependent (exp)	85.4	1.30	1.00	14
Model	T dependent (field)	370.5	1.23	1.00	8
Model	Fixed (1 mg l^−1^)	198.5	1.16	1.00	4

Model results using parameters fitted on the microcosm experiment with temperature dependent oxygen levels. Q_10_ Estimates indicate the reaction rate increase at a 10 degree temperature rise.

Obviously, several other biochemical reactions preceding denitrification may be directly affected by temperature. For instance, higher temperatures may promote the production of ammonium by mineralization and the conversion of ammonium to nitrate by nitrification. On the other hand, increased temperature may indirectly affect denitrification through its effect on other factors, for example by decreasing redox potential and organic carbon availability [3], [14], [15]. In nitrate limited systems direct and indirect effects of temperature on mineralization and nitrification may play a larger role than in this study, as they provide nitrate for the denitrification process. This may work out in different ways as nitrification rates generally increase with temperature [3], while at the same time lowered dissolved oxygen concentrations caused by increased temperature may reduce nitrification rates and thereby reduce denitrification in nitrate limited systems [14].

The dissimilatory reduction of nitrate to ammonium (DNRA), a process that competes with denitrification, will be affected by warming as well. Like denitrification, DNRA occurs under anoxic conditions [16], and increases with warming [17], [18], [19]. Thus, effects of warming on DNRA are likely also amplified by temperature effects on dissolved oxygen. Availability of organic carbon and nitrate will determine whether denitrification or DNRA will dominate in absolute nitrate reduction [20].

This illustrates the complexity of predicting the effect of warming on environmental processes. Counteracting effects may buffer overall temperature effects, while in other situations synergy between positive effects can lead to greatly amplified temperature sensitivity. Our results strongly indicate that the latter is the case for denitrification in freshwater ecosystems like ditches and shallow lakes. The fact that such synergistic temperature effects can build up to a very steep overall temperature dependence has recently also been demonstrated in a study of newly developing ecosystems [21]. While freshwater ecosystems may be particularly sensitive to the effect we describe, a similar synergistic effect of temperature on denitrification has been hypothesized for terrestrial soils [4], [22].

The overall consequences of an alteration of aquatic denitrification with warming are difficult to oversee. Increased denitrification may help to reduce eutrophication effects in shallow lakes and coastal waters. On the other hand, warming may also alter nitrogen loading through changes in mineralization, nitrogen deposition, precipitation and land-use [23]. Thus, although absolute denitrification rates may increase, warming will not necessarily lead to a higher nitrogen removal efficiency. Importantly, greenhouse gas emissions could rise with denitrification rates. Lowered oxygen levels can affect the fraction of N_2_O produced in denitrification and nitrification in various ways [24], [25], [26], making the overall effect of temperature difficult to predict. Nonetheless, as denitrification inevitably produces the greenhouse gases N_2_O and CO_2_ [2], a doubling of denitrification with a 3 degree temperature rise implies a potentially significant positive feedback on global warming [4].

Clearly, we are still far from understanding many aspects of the human alteration of the worlds nitrogen cycle. Nonetheless, our results indicate that denitrification in freshwater ecosystems may be particularly sensitive to warming due to the strong synergistic oxygen effects in these systems.

## Materials and Methods

### Microcosm setup

Two similar microcosm experiments were performed, in the first experiment oxygen concentrations were not controlled, in the second one they were kept below 1 mg l^−1^O_2_. For each experiment we set up 12 microcosms. Each microcosm contained a litre of organic sediment originating from a nearby eutrophic pond, 7 litre of Smart and Barko growth medium containing 1.3 mg N l^−1^ (as NH_4_NO_3_) and 0.19 mg P l^−1^(as K_2_HPO_4_), and 60 gram wet weight of *Elodea nuttallii* (Planch.) St. John, which originated from an experimental drainage ditch (Sinderhoeve experimental station, Renkum, the Netherlands 51°59′55.08″N, 5°45′21.40″E). The microcosms were kept in water baths at 17.5°C at a 12/12 D/L cycle for 5 weeks before the start of the denitrification measurements to allow biofilm development. Twenty hours before the denitrification measurements we applied 4 different temperature treatments (in triplicate) to the microcosms: 10, 15, 20 and 25 °C.

In the experiment with controlled oxygen, water column dissolved oxygen levels in the microcosms were set to <1.2 mg l^−1^ by gently bubbling the water column with helium, as previous tests showed that DO would further drop to 1.0 mg l^−1^ in the 4 hours acclimatization period before the denitrification measurements. When a concentration of 1.2 mg l^−1^ was reached the microcosms were closed by an airtight disc. Denitrification measurements in these microcosms started 4 hours after setting the low oxygen levels.

### Denitrification measurements

Denitrification measurements were performed in the dark after 8 hours of darkness to prevent the production of gas bubbles due to photosynthesis which may disturb the measurements. For the denitrification measurements the microcosms were closed with airtight lids. Each lid had a screw opening for a stirrer, which gently stirred the water continuously to provide mixing of substrates, and a screw cap-opening with a septum. The lids where positioned 4 cm under the water surface. The growth medium under the lids of the microcosms was enriched with 1.16 mg l^−1^ ^15^N and 0.56 mg l^−1^ ^14^N both in the form of NaNO_3_, which was injected through the septum. We added 0.5 mg l^−1^ glucose as a source of easily oxidizable carbon to prevent carbon limitation of the denitrifying bacteria during the denitrification measurements. Water was sampled 0.25, 1, 2 and 3 hours after injection of the ^15^N[Na-NO_3_] solution. Samples (5 ml, in triplicate) were taken through the septum using a 10 ml airtight glass syringe, after which they were injected into 12 ml Exetainers (Labco, high Wycombe, UK). These exetainers contained 100 µl 50% (w:v) ZnCl_2_ solution to stop biological processes in the samples, and were pre-flushed with helium to prevent air contamination of the samples, after which 5 ml of helium was removed to create space for the water sample [27]. Samples were stored at room temperature and before analysis they were vigorously shaken to transfer the dissolved N_2_ into the helium headspace. Denitrification rates were calculated from accumulation of ^14,15^N_2_ and ^15,15^N_2_ in the headspace [28], measured at a SerCon Cryoprep trace gas concentration system interfaced to a PDZ Europa 20-20 isotope ratio mass spectrometer (Sercon Ltd., Cheshire, UK) at the UC Davis stable isotope facility (Davis, CA, USA).

### Field study

A field study was performed in 3 vegetated experimental ditches (de Nieuwlanden experimental station, Wageningen, the Netherlands, 51°58′26.05″N, 5°38′35.02″E.). Measurements were performed weekly between July and August 2001 and 2002, and daily for 20 days in September and October 2002. A split-box measuring device which contained three separate compartments was placed under water over the sediment in order to trap the produced dinitrogen gas. Each chamber was spiked with ^15^N[Na-NO_3_] to reach a concentration of 0.5 to 0.9 mg N l^−1^. The water in the chambers was gently stirred continuously. Water was sampled (5 ml in triplicate) 0.5, 1.5 and 2.5 hours after spiking. Denitrification rates where further determined as described above.

### Model approach

To further explore if the strong effect of temperature on denitrification can be realistically explained by the coupled temperature dependencies of respiration, primary production and oxygen solubility, we formulated a simple model for the dependence of denitrification on temperature and oxygen. We assumed that the temperature effect, for the temperature range between 5 and 25°C, can be described by a modified Arrhenius expression [5], [10], and the effect of oxygen follows inverse Michaelis-Menten kinetics [5]:
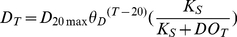
(2)where D_T_ is the denitrification rate in µmol N m^−2^ h^−1^, at temperature T (°C), D_20max_ is the denitrification at 20°C in µmol N m^−2^ h^−1^ in the absence of oxygen, θ_D_ is the temperature coefficient for denitrification under fixed oxygen conditions (dimensionless), K_S_ is the half saturation constant of denitrification for oxygen in mg l^−1^, and DO_T_ is the ambient dissolved oxygen concentration in mg l^−1^ at temperature T(°C). In steady state conditions DO can be described by:
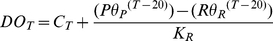
(3)where P is an overall DO-production rate (g m^−3^ d^−1^), θ_P_ is the temperature coefficient of DO-production (dimensionless), R is an overall DO-consumption rate (g m^−3^ d^−1^), θ_R_ is the temperature coefficient of DO-consumption (dimensionless), K_R_ is the re-aeration constant (d^−1^), and C_T_ is the saturation concentration of DO (mg l^−1^) at a certain temperature T (°C) [5] which is quantified as:
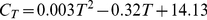
(4)


Parameter values were taken from the literature (θ_P_, θ_R_ and K_R_) [9], [13], [29] and previous experiments in vegetated microcosms (K_s_) [30].The remaining parameters (P, R, D_20max_, θ_D_) were estimated by fitting the model to the experimental and field data, as they are system specific (Table 2). Production (P) should be proportional to plant biomass and vary with plant productivity. The calibrated values for P correspond well to values for oxygen production found in the literature [10], [31]. Respiration (R) will vary with organic matter availability, which in turn depends on long term production and respiration. For R we calibrated different values for the experimental data and for the ditch data (2.3 g m^−3^ d^−1^ and 3.3 g m^−3^ d^−1^), which is likely due to the fact that the microcosm sediments were less organic than the ditch sediments. Still, both calibrated values are in agreement with commonly observed rates of DO-consumption (macrophyte respiration and sediment oxygen demand) in aquatic ecosystems, which range from 0.4–2 g m^−2^ d^−1^ [32], [33]. The calibrated value for D_20max_ is in the range of what might be expected for vegetated drainage ditches [34].

**Table 2:** Model parameter values.

Symbol	Description	Unit	Value	Source
D_20max_	Denitrification at 20 degrees under anoxic conditions	µmol N m^−2^ h^−1^	232; 645	Exp 1; Ditch enclosures
θ_D_	Temperature activity coefficient denitrification	Dimensionless	1.16	Exp 1
K_s_	Half saturation constant of denitrification for oxygen	mg l^−1^	6.0	Exp 1
P	DO Production rate	g m^−3^ d^−1^	1.94	Exp 1
R	DO Consumption rate	g m^−3^ d^−1^	2.28; 3.26	Exp 1; Ditch dataset
θ_P_	Temperature activity coefficient photosynthesis	Dimensionless	1.04	[9], [13]
θ_R_	Temperature activity coefficient respiration	Dimensionless	1.10	[9], [13]
K_R_	Re-aeration constant	d^−1^	0.30	[29]
